# Microscale Contact Electrification with Unprecedented High Intrinsic Charge Density

**DOI:** 10.1002/smll.202506466

**Published:** 2025-08-18

**Authors:** Chaojie Chen, Jinhui Nie, Jie An, Xin Xia, Zhanghui Wu, Hongqiang Wang, Huachen Cui, Quanshui Zheng, Yunlong Zi

**Affiliations:** ^1^ Department of Mechanical and Automation Engineering The Chinese University of Hong Kong Shatin N.T. Hong Kong China; ^2^ Institute of Superlubricity Technology Research Institute of Tsinghua University in Shenzhen Shenzhen 518057 China; ^3^ Department of Engineering Mechanics School of Aerospace Engineering Tsinghua University Beijing 100084 China; ^4^ Thrust of Sustainable Energy and Environment The Hong Kong University of Science and Technology (Guangzhou) Nansha Guangzhou Guangdong 511400 China; ^5^ Department of Mechanical and Energy Engineering Southern University of Science and Technology Shenzhen 518000 China; ^6^ Smart Manufacturing Thrust Systems Hub The Hong Kong University of Science and Technology (Guangzhou) Guangzhou Guangdong 511458 China; ^7^ Center of Double Helix Institute of Materials Research Shenzhen International Graduate School Tsinghua University Shenzhen 518055 China; ^8^ Center for Nano and Micro Mechanics and Applied Mechanics Laboratory Department of Engineering Mechanics Tsinghua University Beijing 10084 China; ^9^ Guangzhou HKUST Fok Ying Tung Research Institute Guangzhou Guangdong 511400 China; ^10^ Division of Integrative Systems and Design The Hong Kong University of Science and Technology Clean Water Bay, Kowloon Tong Hong Kong China

**Keywords:** contact efficiency, contact electrification, triboelectric charge density

## Abstract

Contact electrification (CE) refers to the charge generation of two materials after contact and separation, benefiting applications including electrophotography, electrostatic self‐assembly, and energy harvesting. However, surface asperities lead to a low effective charging area and restrict the net CE charge density. Here, atomically flat polytetrafluoroethylene (PTFE) and graphite microflake are used to increase the contact efficiency and achieve an unprecedented intrinsic CE charge density of 2.6 mC m^−2^. Crucially, the unipolar negative charging region is observed at the graphite/PTFE interface, which effectively eliminates charge cancellation and amplifies net charge density, diverging from the charge mosaic pattern. These findings provide a fundamental understanding for further material/interface development toward controllable triboelectric charge density.

## Graphical abstract

With the atomically flat contact interface by a microscale graphite slider and polytetrafluoroethylene, microscale contact electrification with high contact efficiency is achieved. The developed microscale triboelectric nanogenerator exhibits a record‐high intrinsic triboelectric charge density of 2.6 mC m^−2^, which is attributed to the greatly enhanced contact efficiency (94%).

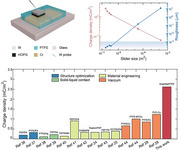

## 1 Introduction

The kinetic energy sources, such as raindrops, random vibration, and human motion, are widely distributed in the surroundings.^[^ ^1^, ^2^, ^3^, ^4^, ^5^, ^6^ ^]^ Scavenging them to power electronic devices is beneficial to alleviate the energy crisis and achieve net‐zero carbon objectives, contributing to the sustainable development of society.^[^ ^7^, ^8^ ^]^ Meanwhile, the rapid development of device miniaturization and nanotechnology shrinks down sensor nodes in both size and power consumption, demanding the development of micro‐ and nano‐scale energy harvesters. These include piezoelectric,^[^ ^9^, ^10^, ^11^ ^]^ electromagnetic, ^[^ ^12^, ^13^ ^]^ and thermoelectric energy harvesters,^[^ ^14^, ^15^ ^]^ which can be integrated into micro sensor nodes to enable self‐powered systems. In them, triboelectric nanogenerators (TENGs) can convert kinetic energies into electricity with original charge generation from contact electrification (CE, i.e., triboelectrification),^[^ ^16^ ^]^ with advantages of high output, low cost, and high compatibility, making it suitable to serve as a sustainable power source for sensor nodes. ^[^ ^17^, ^18^, ^19^ ^]^

For traditional macroscale TENGs (macro‐TENGs), the low energy density caused by the limited charge density severely restricts their application in energy harvesting and powering, especially as compared to solar cells and batteries. The existing high charge densities were nearly all achieved by the charge excitation methods, such as the self‐excitation strategy and the voltage‐multiplying circuit.^[^ ^20^, ^21^, ^22^ ^]^ However, these methods demand excitation circuits, which bring additional power consumption and volume. Other methods, such as material selection/engineering^[^ ^23^, ^24^, ^25^, ^26^, ^27^ ^]^ can enhance its intrinsic CE charge density (ICECD)—which arises solely from contacted materials, without any charge excitation methods. However, even with optimization, these approaches achieve a maximum ICECD of only 1.25 mC m^−2^.^[^ ^28^ ^]^ In fact, the contact state between two surfaces is critical for maximizing the ICECD. Specifically, the surface roughness causes voids and prevents the two surfaces from full contact, resulting in low intrinsic charge generation. In contrast to macroscale surfaces, microscale surfaces with nanoscale roughness are possible to approach full contact by the van der Waals (vdW) interaction. Therefore, developing microscale contact electrification may enable microscale TENGs (micro‐TENGs) to meet the needs of micro/nano energy harvesters, with greatly enhanced ICECD as well as the output performance.

The interface between two objects can include two types of areas^[^ ^29^ ^]^: the apparent contact area *A*_0_ and effective contact area *A_e_*. Specifically, the apparent contact area is the overall size of contact surfaces, while the effective contact area is the sum of contacted areas with tribo‐charge generated. Therefore, *A*_0_ is determined by the geometric size of objects and *A_e_* depends on surface roughness, material properties, and pressure. It should be noted that only *A_e_* is responsible for CE, which demands effective contact of surfaces. However, the surface roughness inevitably brings some issues to deteriorate CE performance. First, *A_e_* is usually much smaller than *A*_0_, resulting in low contact efficiency *η* as the percentage ratio of *A_e_*/*A*_0_ and low ICECD. It was proven that the triboelectric charge density can be maximized by increasing *A_e_* ^30^. However, the contact efficiency is lower than 50%,^[^ ^30^ ^]^ which means that more than half of the full surface area does not contribute to CE. Second, the asperities induced uneven stress distribution can lead to a non‐uniform mosaic distribution of bipolar charges.^[^ ^31^, ^32^, ^33^, ^34^ ^]^ This means that the measured charge transfer only represents the net charge, which is relatively small due to the compensation between positive and negative charges, ultimately resulting in a low surface charge density. Last, discharge can happen in the void region (i.e., non‐contact area) when the surface electric field reaches the breakdown threshold, resulting in additional charge dissipation.^[^ ^35^ ^]^ Thus, CE in microscale surfaces with nanoscale roughness may greatly enhance the ICECD by boosting *A_e_*, facilitating uniform charge distribution, and avoiding void regions.

Herein, we design a micro‐TENG to improve the contact efficiency between triboelectric pairs and achieve an unprecedented ICECD of 2.6 mC m^−2^ that is 41 times higher than the macroscale device. The micro‐TENG features a sliding freestanding triboelectric‐layer (SFT) structure including a graphite microflake slider and polytetrafluoroethylene (PTFE) as the stator. Its output performance is characterized by investigating the influence of various parameters, including slider size, frequency, and PTFE thickness. Moreover, the scaling effect of ICECD is investigated. It is found that the effective contact area contributes to the CE process, in which the mechanism is strongly related to the repulsive vdW interaction in the microscale. This work explores the maximum ICECD of PTFE, discloses the mechanism in microscale, and explains the scaling effect, which broadens our understanding of the CE mechanism and helps to develop high‐performance triboelectric devices.

## 2 Results and Discussion

### 2.1 Micro‐TENG Design and Fabrication

**Figure 1:**
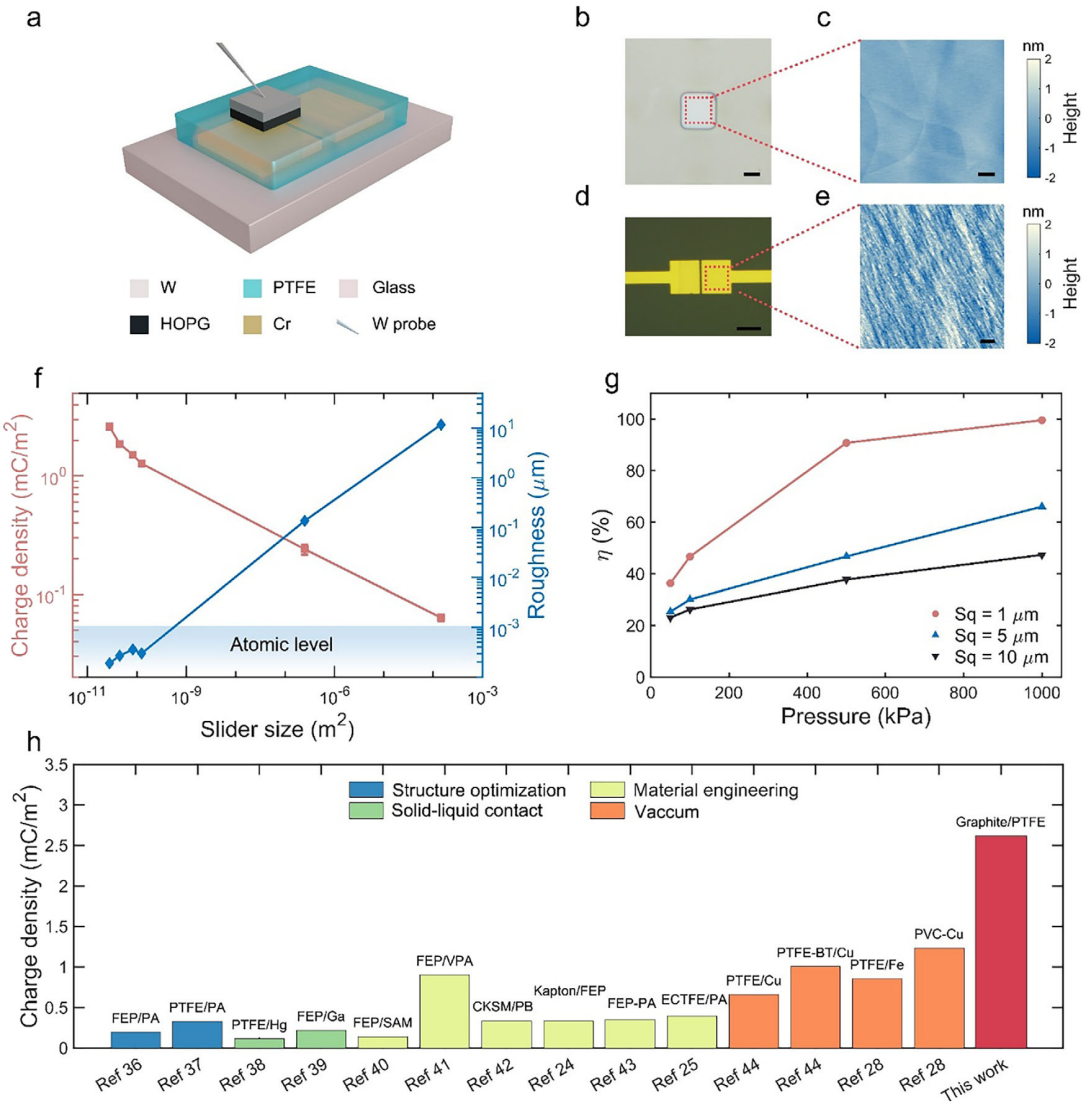
Promotion of ICECD by micro‐TENGs. a) Schematic diagram of micro‐TENG structure. b) Photo of the bottom side of the slider. Scale bar, 5 µm. c) Surface morphology of the bottom side of the slider, and the roughness is 0.3 nm. Scale bar, 1 µm. d) Photo of the micro‐TENG stator. Scale bar, 20 µm. e) Surface morphology of PTFE, and its roughness is ≈0.8 nm. Scale bar, 1 µm. f) Scaling effect of the ICECD. g) Simulation results of the relationship between contact efficiency and roughness under different loading pressures. h) Comparing the ICECD of materials among previous works^[^ ^24^, ^25^, ^28^, ^36^, ^37^, ^38^, ^39^, ^40^, ^41^, ^42^, ^43^, ^44^ ^]^ and this work.

The micro‐TENG is composed of a glass substrate, chromium (Cr) electrode, PTFE dielectric layer, and graphite micro slider, with the slider size varied from 5.8 to 11.6 µm, as presented in **Figure** **1**a. Graphite slider includes tungsten (W) overlay and highly oriented pyrolytic graphite (HOPG). The device was fabricated using micro/nano processing technique with details in Methods and Figures S1–S8 (Supporting Information). The roughness of the graphite micro slider is 0.3 nm (Figure 1b,c), while that of the PTFE is 0.7 nm (Figure 1d,e). Therefore, these two flat surfaces may interact with each other via the vdW force due to the atomic‐level roughness, which is close to full contact. Macro‐TENGs were prepared using the same triboelectric materials of graphite and PTFE, with the size of 0.5 mm (Figure S9a,b, Supporting Information) and 12 mm (Figure S10a,b, Supporting Information), respectively. By comparing the micro‐ and macro‐TENGs, the measured ICECD generated by graphite and PTFE increases as the device size decreases and the surface roughness decreases, demonstrating the scaling effect as shown in Figure 1f. Especially for micro‐TENGs, even though the surface roughness remains at the atomic level, the ICECD also increases with the decrease of the device size. The ICECD of the smallest device (28 µm^2^) is 2.6 mC m^−2^, which is 41 times larger than that of the largest device (144 mm^2^). The device with 0.25 mm^2^ *a*rea shows the low surface undulation within 1 µm (Figure S9c, Supporting Information), while the fluctuation of the largest one (144 mm^2^) *i*s up to 50 µm (Figure S10c, Supporting Information). To qualitatively analyze the influence of roughness on the contact efficiency η, a finite element method (FEM) was used. With the loading force applied on one contacted surface with varied surface roughness, the finite elements in the other contacted surface that are displaced along the force direction are counted as the effective contact regions. The FEM results demonstrate that the η increases when the surface roughness decreases or the loading force increases, as shown in Figure 1g, contributing to the ICECD, with the simulation method specified in Method and Figure S11 (Supporting Information). Overall, CE through micro‐TENGs with low surface roughness demonstrates unprecedented ICECD. In previous works, the ICECD can be boosted by structure optimization, material engineering, solid‐liquid contact, vacuum, high pressure, etc., with the highest value of only 0.85 mC m^−2^ achieved for PTFE. Compared with them, our micro‐TENG promoted the record of the ICECD by over three times (Figure 1h).

### 2.2 Influential Factors on the ICECD

**Figure 2:**
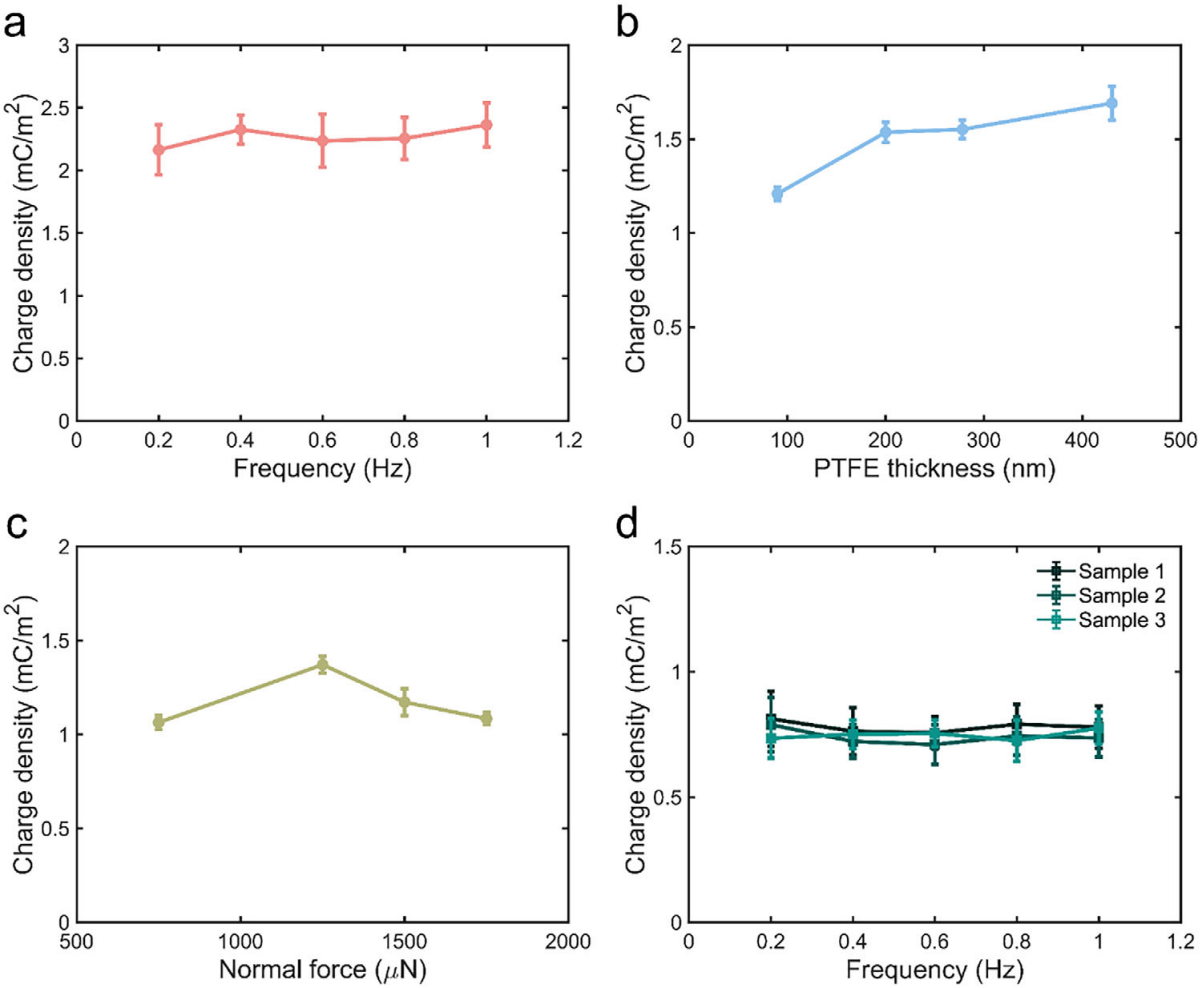
Output performance of micro‐TENGs. a) Charge density influenced by the frequency from 0.2 Hz to 1 Hz. The slider width is 5.8 µm, and the thickness of PTFE is 220 nm. b) Charge density generated by PTFE with different thickness. c) Charge density obtained under different normal forces. The sliding frequency and slider size in (b) and (c) are 1 Hz and 11.6 µm, respectively. d) Charge density of SiO_2_‐based micro‐TENG. The slider width is 7.1 µm.

**Figure 3:**
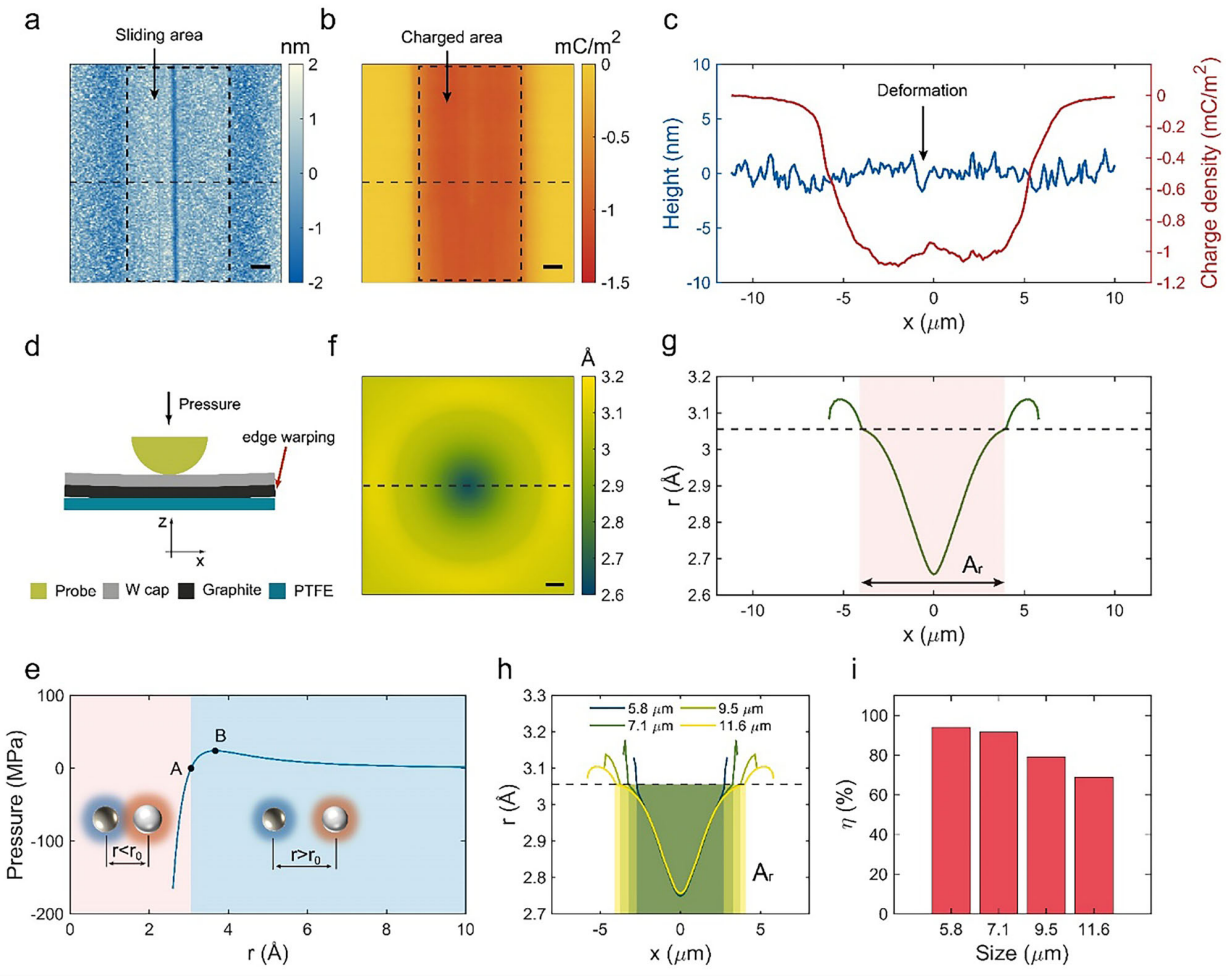
Contact electrification in microscale. a) Surface morphology and b) charge density distribution of PTFE after friction. Scale bar, 2 µm. c) Cross‐section profile extracted from (a) and (b). d) Schematic of FEM model. e) The relationship between the vdW pressure and distance for graphite/PTFE contact. f) Simulated relative distance *r* distribution on the contact surface. Scale bar, 1 µm. g) Extracted distance change between the graphite and PTFE along the x direction from (f). The middle shallow pink area is the *A*_e_ that contributes to CE. Simulation results of h) distance change and i) contact efficiency η for graphite/PTFE contact. The contact widths are 5.8, 7.1, 9.5, and 11.6 µm. The original point *x*   =   0 in (c), (g), and (h) is set at the contact point between probe and PTFE.

Experiments were conducted to characterize micro‐TENGs’ charge density and investigate the influence of frequency, PTFE thickness, and loading force. As shown in **Figure** **2**a, charge density is almost independent of the frequency change, which varies in the range of 2.1 to 2.6 mC m^−2^. For the influence of thickness, the charge density slightly increases when the thickness of PTFE increases (Figure 2b), which can be due to the suppressed charge leakage (Figure S12, Supporting Information).^[^ ^45^ ^]^ For the influence of loading force, the charge density first increases and then decreases as the force continuously increases (Figure 2c). This may be related to the edge warping of graphite.^[^ ^46^ ^]^ The point‐surface contact between probe and graphite may lead to stress concentration that separates the edge part of graphite from the PTFE substrate (Figure 3d), reducing the contact area and charge density. Relative electric signals in the time domain are provided in Figures S13–S15 (Supporting Information). By replacing the PTFE with silicon oxide, the pair of SiO_2_ and graphite shows a high charge density of 0.75 mC m^−2^ (Figure 2d) that is ten times higher than other inorganic materials,^[^ ^47^ ^]^ and comparable to fluoropolymers in Figure 1h.

### 2.3 Contact Electrification Mechanism in Micro‐TENGs

To understand the underlying mechanism for high ICECD, the charge distribution of the sliding area by the graphite flake (width:11.6 µm) was characterized by kelvin probe force microscope (KPFM), with the center of the sliding trajectory shown in the morphology picture (**Figure** **3**a), and the charge density distribution (Figure 3b) calculated from potential distribution obtained in KPFM image (Figure S16, Supporting Information). The transferred charge density Δσ was extracted from the potential distributions using Equation (1),

(1) $$\Delta \sigma =\frac{\Delta V\varepsilon_{0}\varepsilon_{PTFE}}{t_{PTFE}}\;$$

where Δ*V* is the potential difference between the charging area and the non‐charging area, ɛ_0_ is the vacuum dielectric constant, ɛ_*PTFE*_ is the relative permittivity of PTFE, *t_PTFE_* is the thickness of the PTFE layer. Detailed derivation can be found in Note S1 (Supporting Information). Before tribocharging, the PTFE surface shows relatively neutral potential (−0.8 V in Figure S17a, Supporting Information), while the charged area shows more negative potential down to ‐5 V after using a graphite slider to rub PTFE (Figure S17b, Supporting Information), showing significant tribocharging behavior. Notably, the charging area in Figure 3b exhibits a uniformly negative surface charge. Unlike the charge mosaic pattern formed by the simultaneous distribution of positive and negative charges^[^ ^31^ ^]^ due to varied surface composition and air breakdown discharge,^[^ ^32^ ^]^ there is no positive charge region present here. The full contact between graphite and PTFE in microscale with uniform composition eliminates the air gap and discharge, resulting in unipolar charging. The unipolar charging in micro‐TENG amplifies the net negative charge generation as there are no positive CE charges to cancel negative charges, making it possible to approach the maximum ICECD. By extracting the cross‐section profile of the morphology, it is found that the middle trench of the sliding area experiences a relatively large deformation (≈1.5 nm, blue line in Figure 3c), attributable to stress concentration during normal force loading by the probe. The CE charge in the charging area gradually decreases over time through diffusion,^[^ ^48^ ^]^ ultimately retaining 30% after 1080 min (Figure S18, Supporting Information). To understand the effective contact area *A*_e_, a FEM model based on a probe pressing a W/graphite flake on the PTFE was used to simulate the contact state between the graphite slider and PTFE, with the cross‐section view of the model shown in Figure 3d. The vdW force between the graphite and PTFE was derived from the Lennard‐Jones (LJ) potential, based on the graphite/PTFE distance *r* extracted from the model (Note S2, Supporting Information). As shown in Figure 3e, the balance point A is at *r*_0_ = 3.05Å and the max adhesive pressure (*P_max_* =  20 MPa) occurs at point B (*r* = 3.67Å). It is noted that the vdW force can be negligible when the distance is larger than 10Å. Figure 3f shows the contact distance on the PTFE surface, which is smaller in the middle area than that in the edge area (see FEM details in Note S3, Supporting Information). In Figure 3g, due to the variation of *r*, the line in Figure 3f can be divided into two parts, including the repulsive vdW force area *A_r_* and attractive vdW force area *A_a_*. As compared with the charge density curve in Figure 3c, it is found that the area with the concentrated charge density almost overlaps with *A*_r_, which are both from ≈−5 µm to ≈5 µm. Previous works have proven that almost all of the CE charge was induced when the atomic force microscope (AFM) tip works on the repulsive region where the electron cloud of materials overlaps.^[^ ^49^, ^50^ ^]^ Meanwhile, it is verified using an AFM tip to interact with PTFE that CE happens within the repulsive region (Figure S19, Supporting Information). Based on these results, it is reasonable to conclude that the area *A_r_* dominates the CE charge generation through the repulsive vdW interaction, which can be considered as effective contact area *A*_e_. To explain the scaling effect at the microscale, the four devices with varied slider sizes were simulated, as presented in Figure 3h. The η increases as the slider width decreases (Figure 3i), and the maximum value is achieved at 94% for the smallest slider size (5.8 µm). Therefore, the trend of η is consistent with the experimental results in Figure 1f.

### 2.4 Voltage and Output Energy Density of the Micro‐TENG

**Figure 4:**
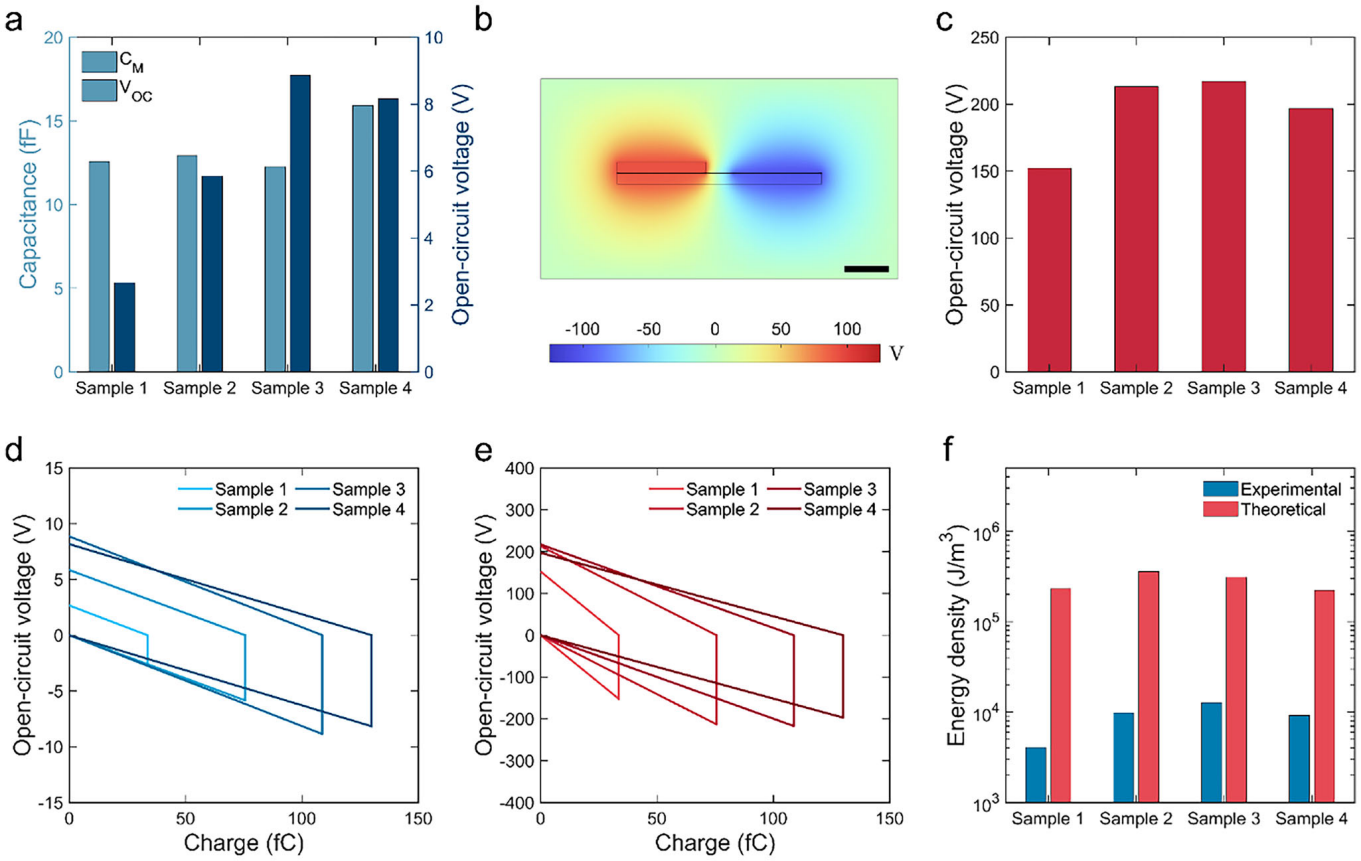
Voltage and energy density of micro‐TENG. a) Capacitance and calculated voltage of micro‐TENGs. b) Voltage simulation of Sample 4. Scale bar, 5 µm. c) Summarized voltage simulation results of four samples. V‐Q plots using d) experimental data and e) simulation data. f) Experimental and simulation energy density of micro‐TENGs. The widths of Sample 1–4 are 5.8, 7.1, 9.5, and 11.6 µm.

The energy density of TENGs is important for applications. To evaluate it, the voltage was first measured. Considering the direct measurement of the open‐circuit voltage (*V*_OC_) may be influenced by the capacitance of the electrometer, as compared to the ultralow internal capacitance of down to 10^−16^ F in the micro‐TENG (Note S4, Supporting Information), we designed a new method to estimate its *V*_OC_. We measured the capacitance of micro‐TENG, which is ≈12 fF (**Figure** **4**a). And then we may evaluate the *V_OC_* using Equation (2).

(2) $$V_{OC}=\frac{Q_{SC}}{C_{M}}\;$$

where *Q_SC_* is the charge transfer in the short‐circuit condition, *C_M_* is the measured capacitance. Here, four samples with different sizes (5.8, 7.1, 9.5, and 11.6 µm) was sellected for analysis. By substituting relative parameters into Equation (2), the *V_OC_* of four samples is within 10 V (Figure 4a). In the meantime, we conducted FEM simulation of the *V*_OC_ that is up to 220 V (Figure 4b,c), which is much larger than the measured value, possibly due to the parasitic capacitance during the measurement. To evaluate the output energy density *E* of the micro‐TENG, the corresponding V‐Q plots are presented in Figure 4d,e. The *E* is estimated by the following equation, without considering the breakdown discharge, which may unlikely happen in micro‐TENG due to the limited voltage:

(3) $$E=\frac{V_{OC}Q_{SC}}{v}\;$$

where *v* is the volume of micro‐TENGs. Finally, the output energy density of micro‐TENGs is in the range of 4.0 × 10^3^ − 1.3 × 10^4^ J/m^3^ through the measured *V*_OC_ (Figure 4f), which is higher than many macro‐TENGs^[^ ^51^ ^]^. However, the output energy density limit of TENGs with charge stimulation may be achieved 10^5^ J/m^3^ level,^[^ ^35^ ^]^ still leaving plenty of room for optimization of the micro‐TENGs. The gap between experimental and theoretical output energy densities is possibly due to the parasitic capacitance effect. Specifically, the measured capacitance is at 10^−14^ F level, while the calculated capacitance is at 10^−16^ F level. This difference may be caused by the parasitic capacitance introduced by the measurement setups (Figure S4a, Supporting Information) and circuits (Figure S8, Supporting Information). Furthermore, the peak power density can achieve 47 W m^−3^ when the load resistance is 10^12^ Ω (Figure S20, Supporting Information).

## 3 Conclusion

In conclusion, this work utilized sub‐nanoscale flat surfaces to approach full contact and investigate the CE phenomenon at the microscale using experimental and theoretical methods. The micro‐sized graphite flake and PTFE showed an unprecedented ICECD of 2.6 mC m^−2^, which is 41 times higher than the control sample in the macroscale. The influences of slider size, dielectric thickness, and frequency on the charge density were investigated. Meanwhile, the high contact intimacy and uniform charge distribution were confirmed by the KPFM results, including the surface morphology and charge density mapping. By simulating the vdW interaction between graphite and PTFE, we found that the contact area was divided into a repulsive‐force region and an attractive‐force region because of the stress concentration. Since the repulsive vdW interaction is mainly responsible for the CE charge generation, the repulsive‐force region can be regarded as the effective contact area. Therefore, the contact efficiency η, which represents the effective area ratio involved in the CE process from macroscale to microscale, elucidates the scaling effect of ICECD. It should be noted that the highest contact efficiency of 94% was realized by the device with 28 µm^2^ area, which is close to full contact. The output energy density of micro‐TENG is in the range of 10^3^ − 10^4^ J/m^3^. This work greatly improves contact efficiency and state‐of‐the‐art ICECD, offering valuable insights into CE behavior at the microscale. By utilizing the advanced in‐situ characterization techniques, it is possible to disclose underlying mechanisms behind CE and provide more insights about long‐standing unsolved issues of triboelectric charging in future works.

## 4 Experimental Section

### Fabrication of Micro‐TENG Stator

The fabrication process is shown in Figure S1 (Supporting Information). First, a 4‐inch glass wafer with a thickness of 500 µm was cleaned. Next, using the double‐layer lift‐off technique to deposit the Cr electrode with a thickness of 8 nm (Figure S2, Supporting Information). Then, depositing Cr (30 nm) and Au (200 nm) on the pad area for wire‐bonding. Finally, dicing the glass wafer (Figure S3, Supporting Information) into 12 mm‐by‐6 mm pieces. The Au pad was covered by Kapton tape as a mask during the deposition process. Magnetron sputtering was used to deposit a PTFE layer on the Cr electrode. By controlling the time (2000, 4000, 6000, and 8000 s), PTFE layer with different thicknesses was obtained (90, 200, 278, and 430 nm), as shown in Figure S4 (Supporting Information).

### Fabrication of Graphite Micropillar Array

The processing flow is presented in Figure S5 (Supporting Information). First, dissociating the highly oriented pyrolytic graphite (HOPG, ZYB grade, Bruker) to obtain a clean surface. Next, sputtering Ti (50 nm) on the surface of HOPG as the adhesion layer and subsequently depositing W (200 nm) and Pt (100 nm) onto the Cr layer. The W layer can support the graphite slider during the reciprocating motion because of its high Young's modulus. Then, coating photoresist on the top of the overlay and patterning it using direct laser writing. Moreover, using ion beam etching to remove metal layers and reactive ion etching to remove the graphite with a depth of 1 µm. Finally, the graphite micropillar array was obtained by removing the photoresist.

### Transfer of Graphite Microflake Slider

The micro graphite slider was pushed out from one graphite micropillar using a tungsten probe (ST‐20‐2.0, GGB Industries Inc.), as demonstrated in Figure S6 (Supporting Information). The sliding surface (contact area) was characterized by an atomic force microscope (Cypher ES, Asylum Research). The photomask was designed to fabricate the micropillar array with the widths of 4, 6, 8, and 10 µm. The obtained slider area is larger than the design because of the lithography error (Figure S7, Supporting Information).

### Measurement and Characterization

The obtained device was connected with an external circuit via gold wire bonding for testing (Figure S8, Supporting Information). The sliding movement of micro‐TENGs was controlled by the piezoelectric stage, and the electric output of micro‐TENG was measured by Keysight B2980. The capacitance was measured by a Keithley 4200A‐SCS parameter analyzer, where a two‐wire sensing method and a metal shielding box were used. The Cypher ES atomic force microscopy was used to obtain the morphology of the slider and surface potential of the PTFE layer. The stepper was used to measure the thickness of the dielectric layer.

### Finite Element Simulation

The influence of roughness on contact efficiency η was simulated using Abaqus/CAE 2024. Square rough surfaces (*Sq*  =  1,  5,  10 µm) with 1 mm width were generated by the Random Surfaces of COMSOL Multiphysics 6.2. The roughness model was transferred to Abaqus/CAE for analysis using the implicit solver. The effective contact area is the sum of substrate elements whose displacement direction is the same as the loading force. The vdW interaction between graphite and PTFE was also investigated by Abaqus/CAE 2024; details can be found in Note S2 (Supporting Information). The *V_OC_* of micro‐TENG was simulated using COMSOL Multiphysics 6.2.

## Conflict of Interest

The authors declare no conflict of interest.

## Author Contributions

C.C., J.N., and Y.Z. conceived the idea. C.C. and J.N. designed the experiments. C.C. carried out the experiments, analyzed the results, and discussed with J.N. and Y.Z. J.A. helped with the measurement setup. X.X. helped with macro‐TENG measurement. W.Z. helped with the FEA simulation. C.C. wrote the paper. C.C., J.N., H.C., Q.Z., and Y.Z. discussed and edited the manuscript. J.N. and Y.Z. supervised the project. All authors reviewed the manuscript.

## Supporting information



Supporting Information

## Data Availability

The data that support the findings of this study are available from the corresponding author upon reasonable request.
