# Supplementary material for: Microscale Contact Electrification with Unprecedented High Intrinsic Charge Density
Source: Small. 2025 Aug 18;21(39):e06466. doi: 10.1002/smll.202506466 (PMC12490182; doi:10.1002/smll.202506466)
Supplement: Supplementary file 1 — Supporting Information [file SMLL-21-e06466-s001.pdf]

NANO · MICRO

small

Supporting Information

for *Small*, DOI 10.1002/smll.202506466

Microscale Contact Electrification with Unprecedented High Intrinsic Charge Density

Chaojie Chen, Jinhui Nie, Jie An, Xin Xia, Zhanghui Wu, Hongqiang Wang, Huachen Cui, Quanshui Zheng and Yunlong Zi**

Supporting Information

Microscale Contact Electrification with Unprecedented High Intrinsic Charge Density

Chaojie Chen, Jinhui Nie*, Jie An, Xin Xia, Zhanghui Wu, Hongqiang Wang, Huachen Cui, Quanshui Zheng, Yunlong Zi*

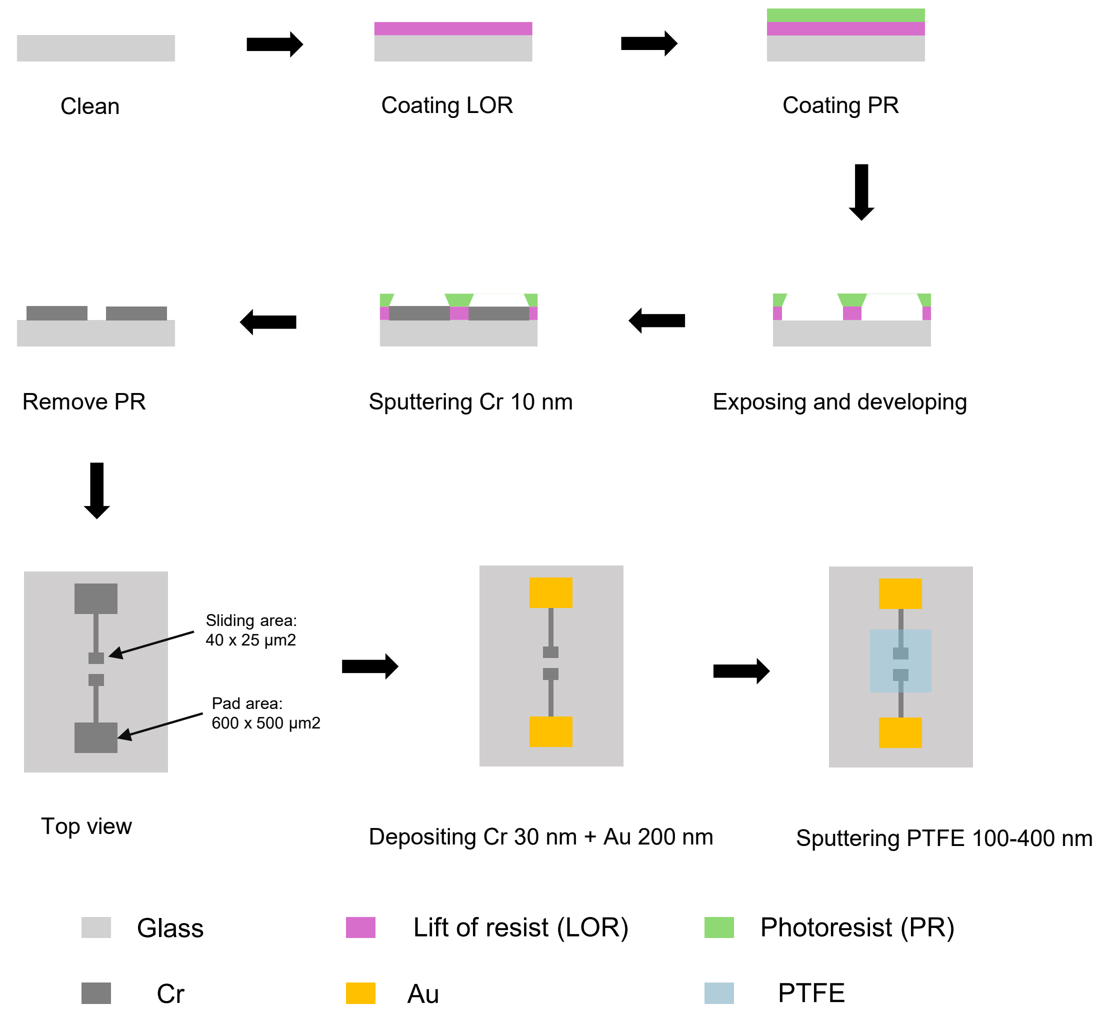

Figure S1. Fabrication of micro-TENG stator.

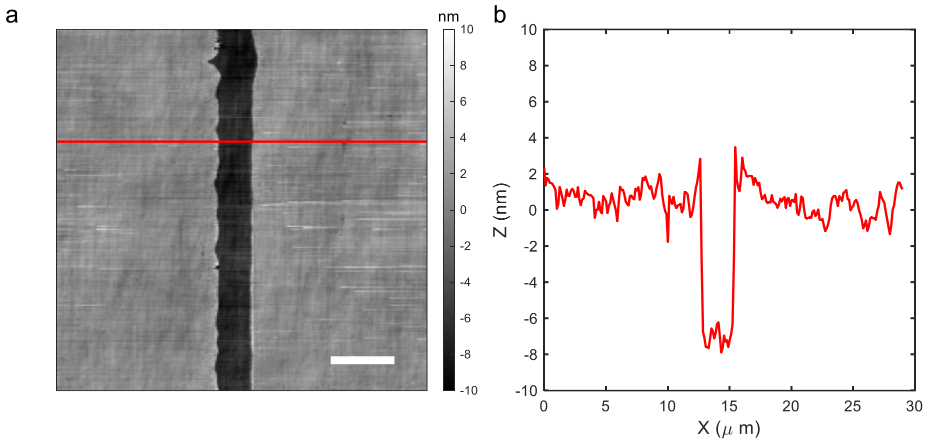

Figure S2. a) Surface morphology. Scale bar, 5 μm. b) Cross section profile.

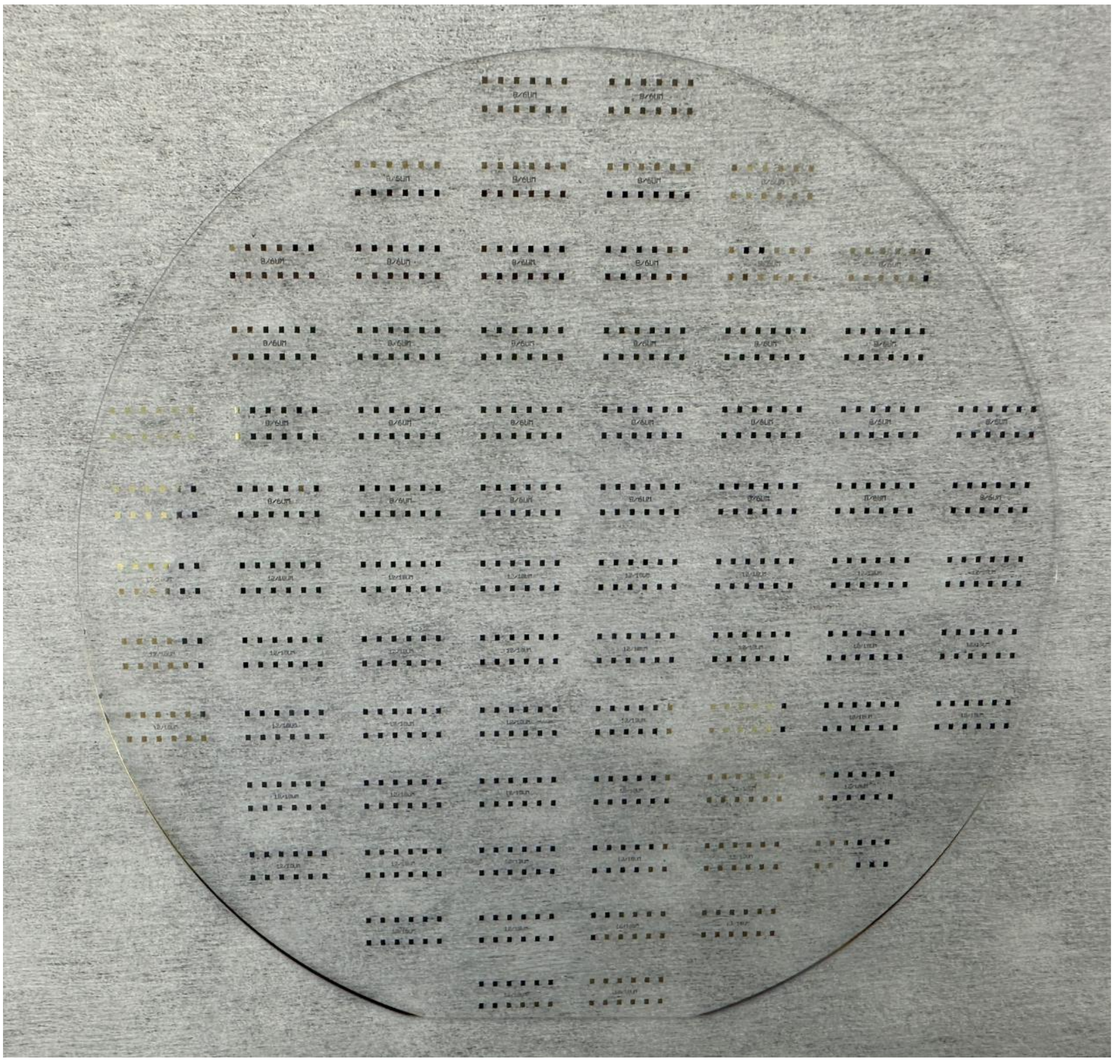

Figure S3. Micro-TENG chip in 4-inch glass wafer.

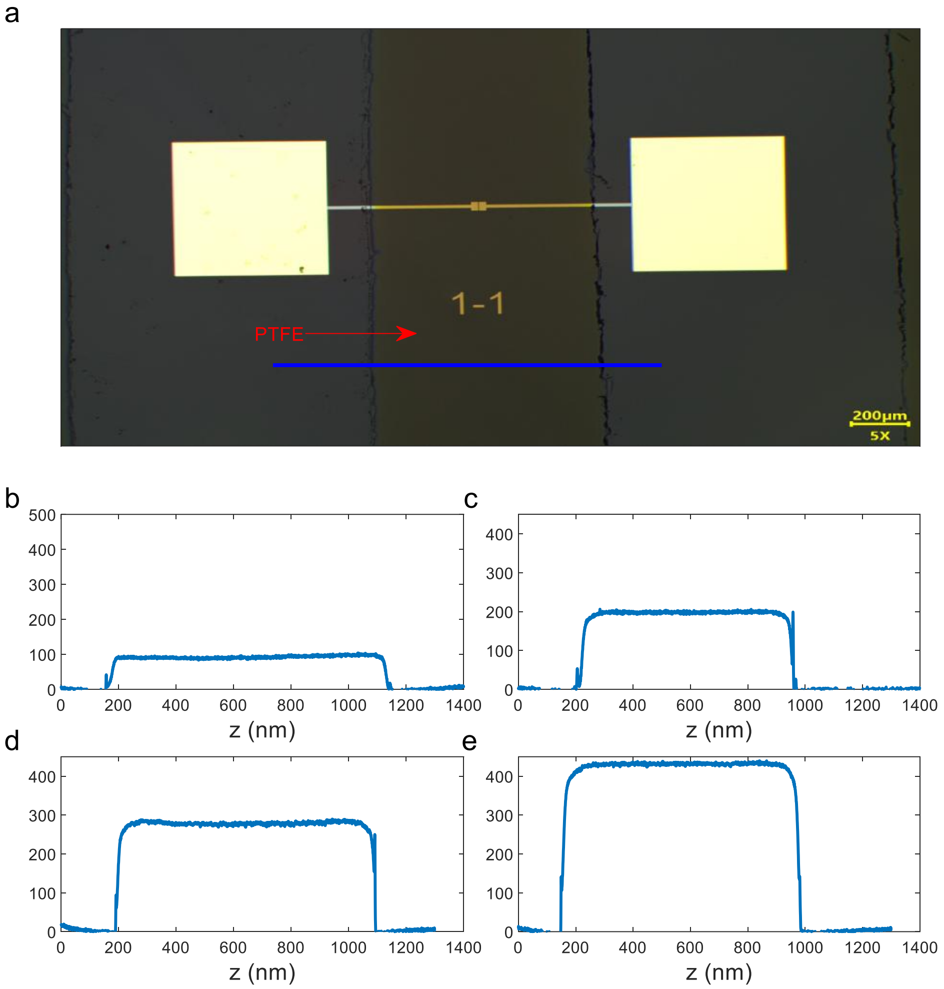

Figure S4. a) Optical image of sample. The Kapton tape is used to cover the Au pad during the sputtering process, and it will be peeled off after processing. The sputtering time is controlled to obtain samples with different PTFE thickness. b) 2000s, 90 nm. c) 4000s, 200 nm. d) 6000s, 278 nm. e) 8000s, 430 nm.

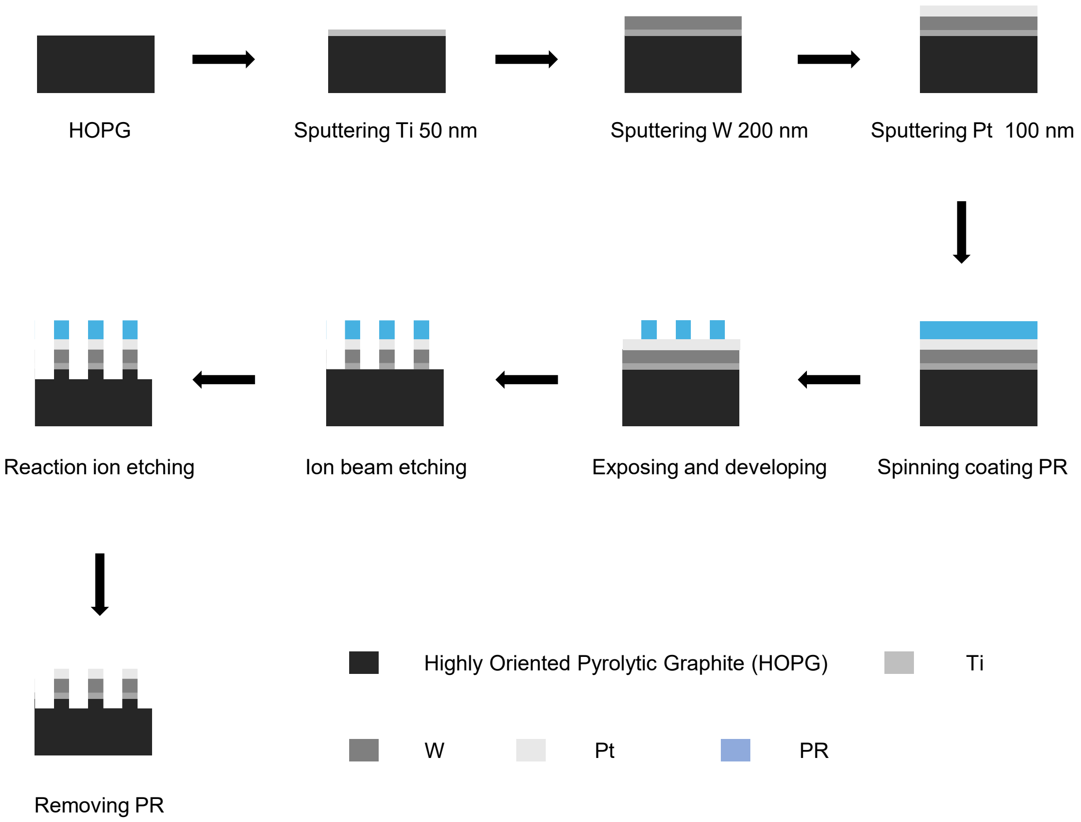

Figure S5. Fabrication process of graphite micropillar array.

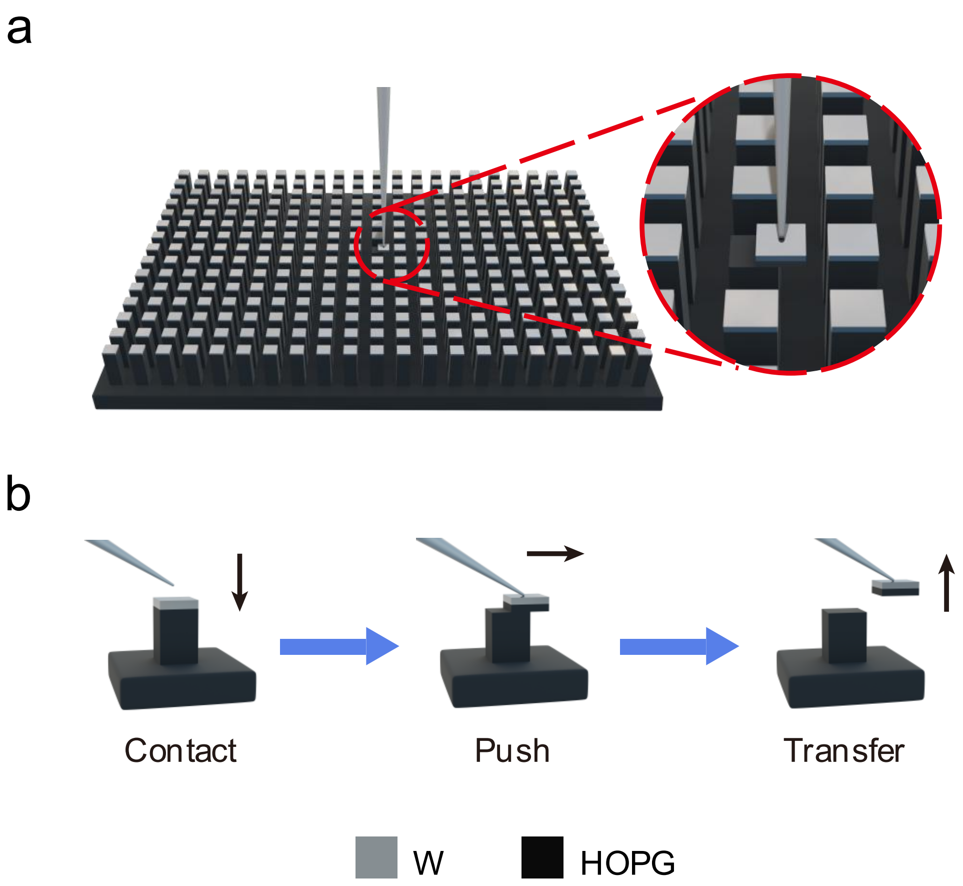

Figure S6. Transferring graphite microflake as the micro-TENG slider.

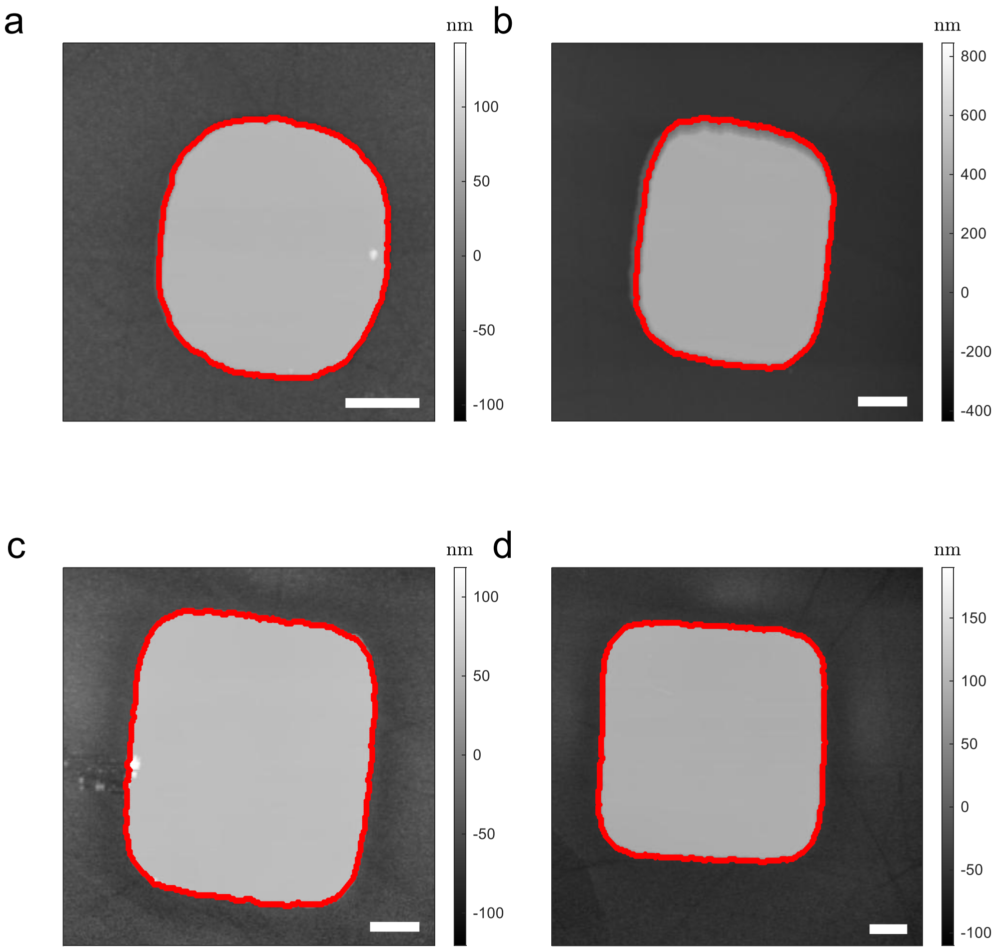

Figure S7. The surface area of different micro-TENG sliders. a) 28 μm^2. b) 45 μm^2. c) 83 μm^2. d) 125 μm^2. Scale bar, 2 μm. The original sizes in mask are 4×4 μm^2, 6×6 μm^2, 8×8 μm^2, 10×10 μm^2. Because of the ultraviolet lithography error, the obtained sample size becomes larger.

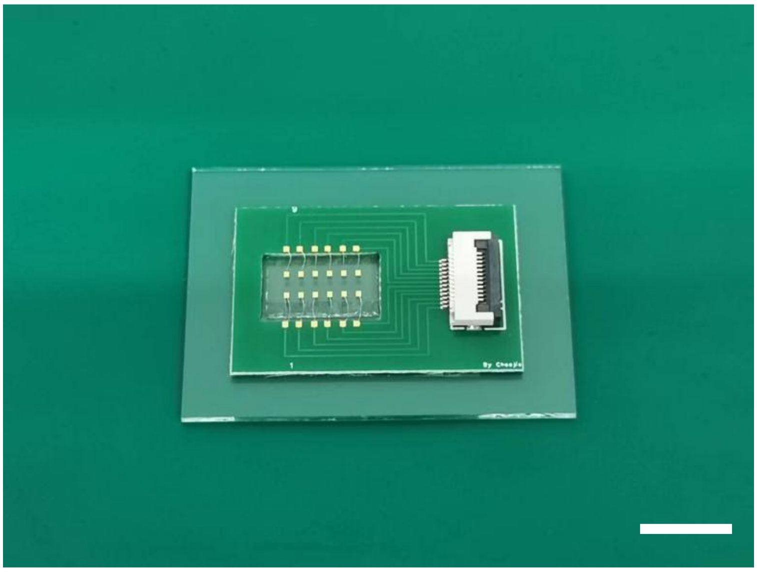

Figure S8. Measurement setup for micro-TENG. The sample is bonded on a printed circuit board (PCB) via Au wire bonding. Scale bar, 1cm.

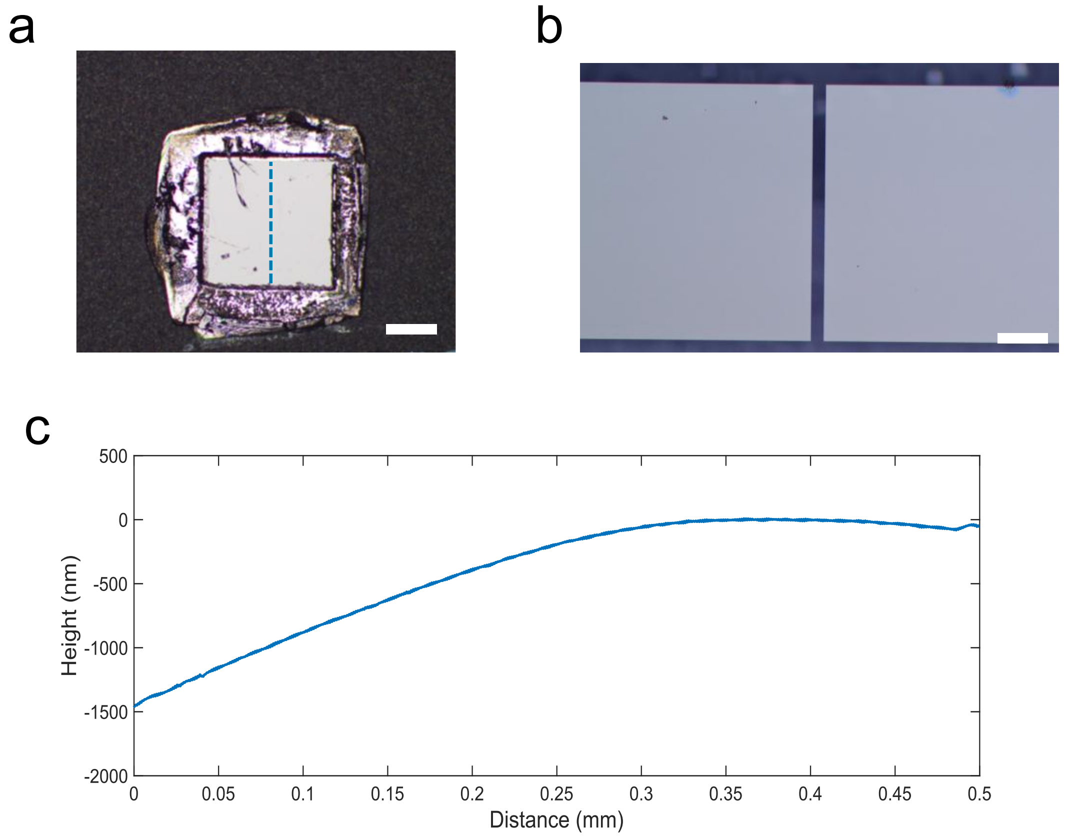

Figure S9. The structure of $\mathbf{0.5 \times 0.5}\ \boldsymbol{mm^2}$ macro-TENG. a) HOPG slider with the size of 0.5×0.5 mm^2. Scale bar, 200 μm. b) The macro-TENG stator. Scale bar, 200 μm. c) Surface profile of the blue line in (a).

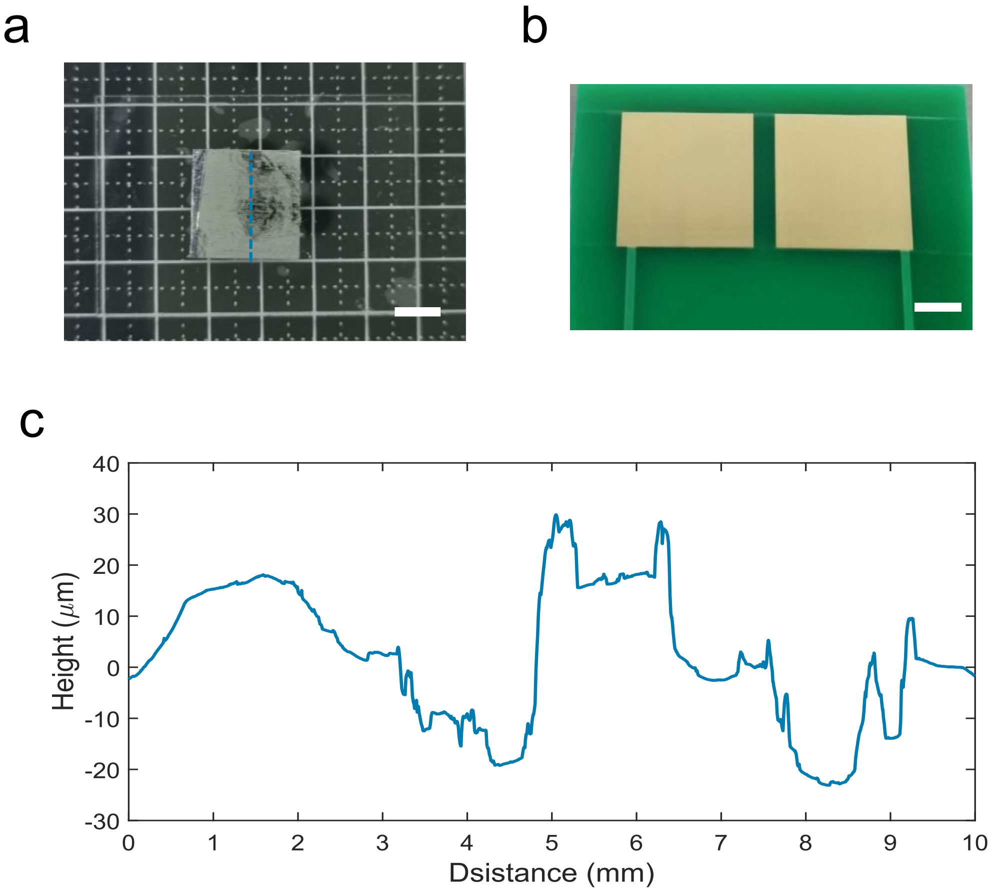

Figure S10. a) HOPG slider with the size of 12×12 mm^2. b) Optical image of stator. The macro-TENG stator is composed of gold electrode and PTFE layer. Scale bar, 5mm. c) Surface profile of the blue line in (a).

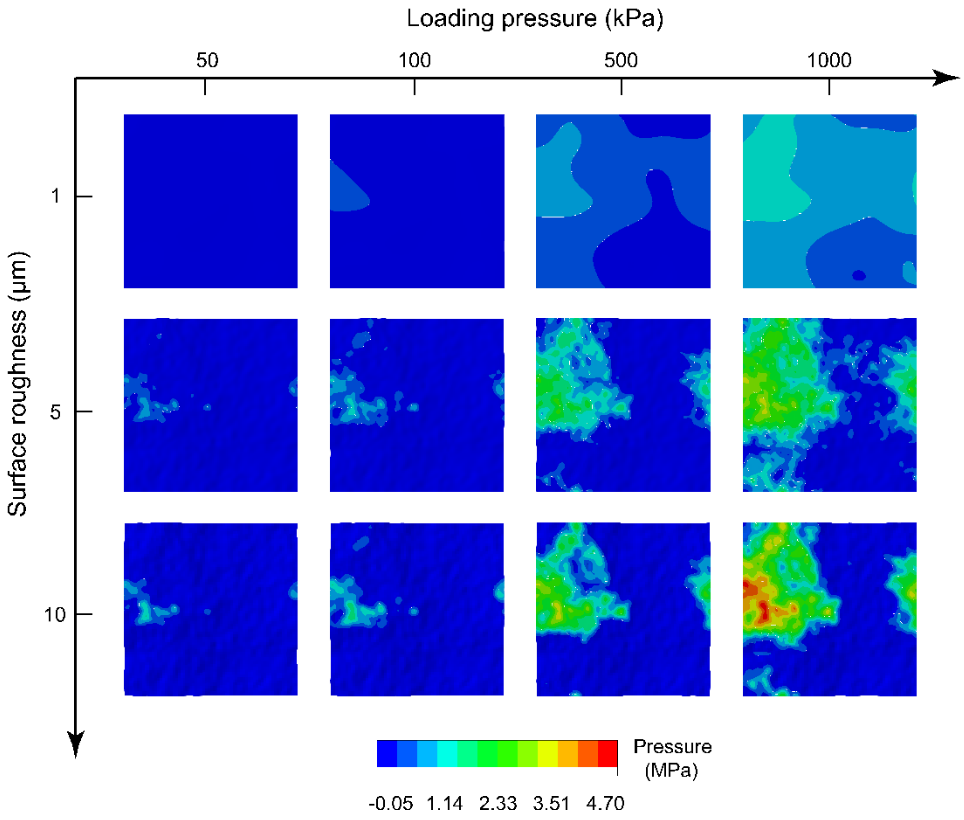

Figure S11. The influence of surface roughness and loading pressure on macroscale contact efficiency η.

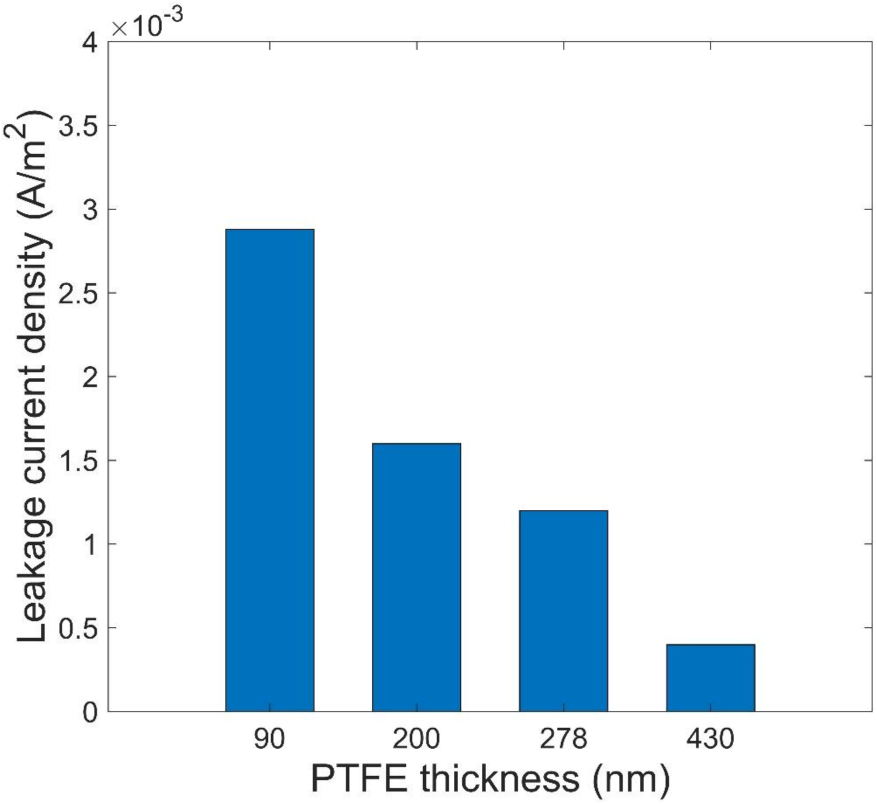

Figure S12 Leakage current density of PTFE with different thicknesses under a 10 V voltage.

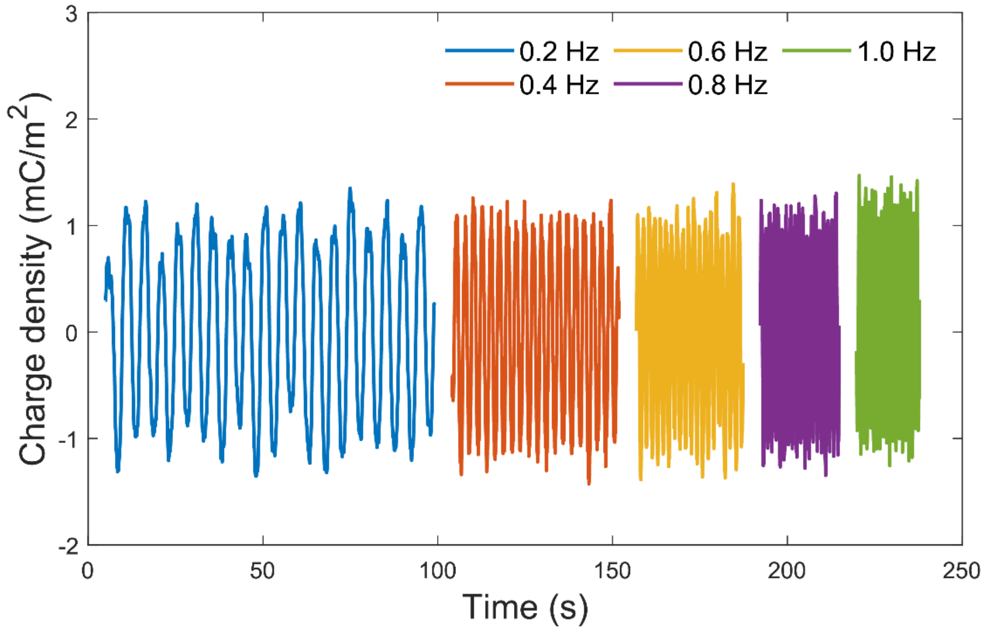

Figure S13. The frequency influence on the electric output of micro-TENG.

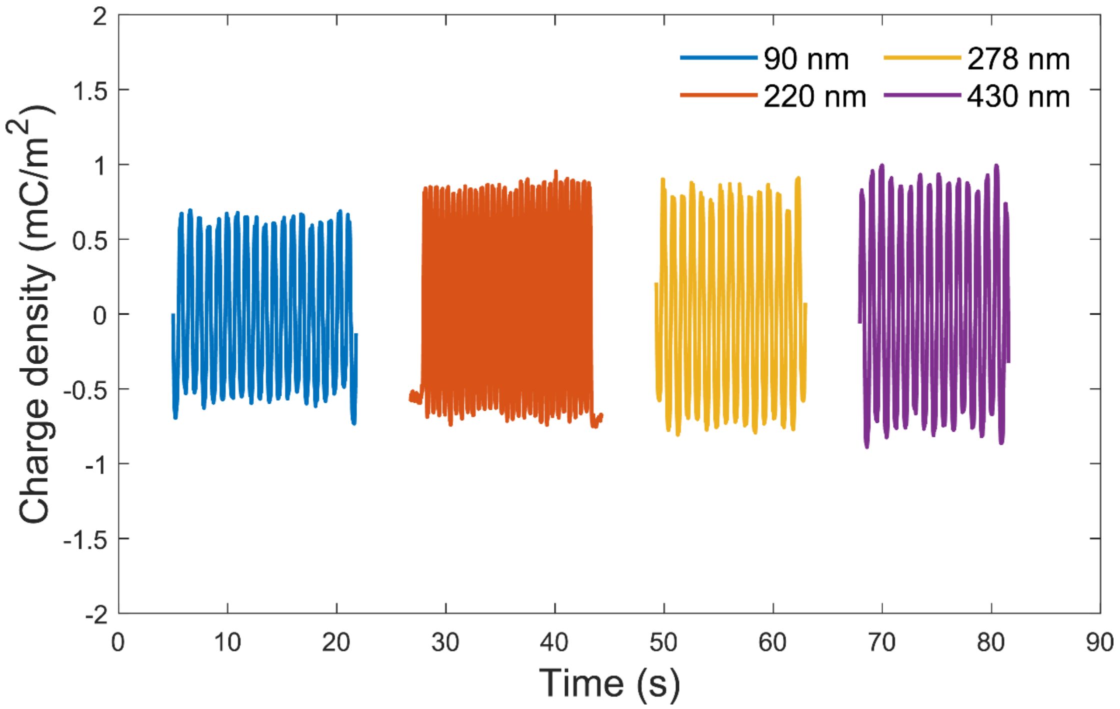

Figure S14. The influence of PTFE thickness on the electric output of micro-TENG.

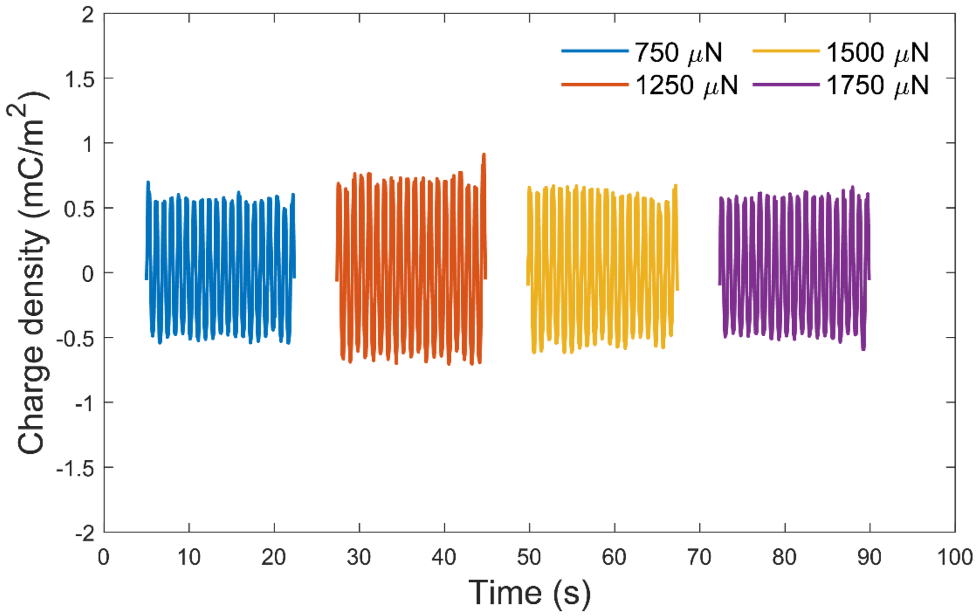

Figure S15. The influence of loading force on the electric output of micro-TENG.

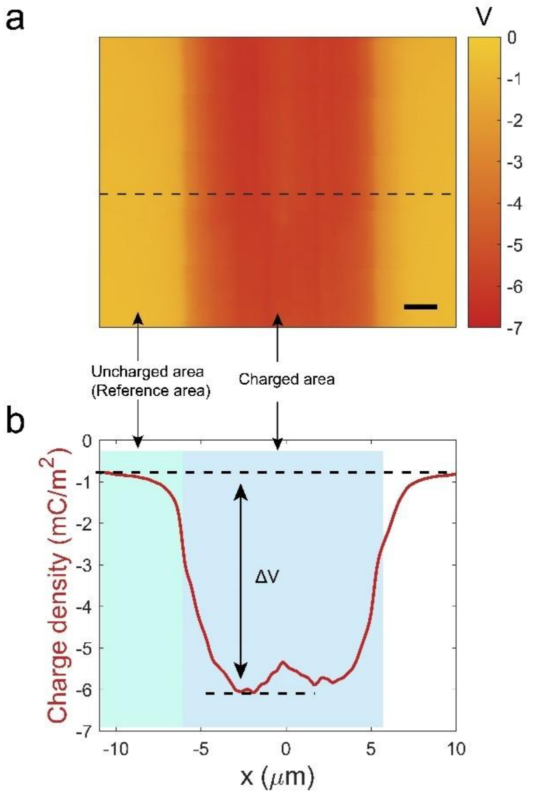

Figure S16. a) Surface potential of charged PTFE including charged area and uncharged area. Scale bar, 2 μm. b) Extracted surface potential profile from (a). The surface potential between charged area and uncharged area is used to calculate the charge transfer density.

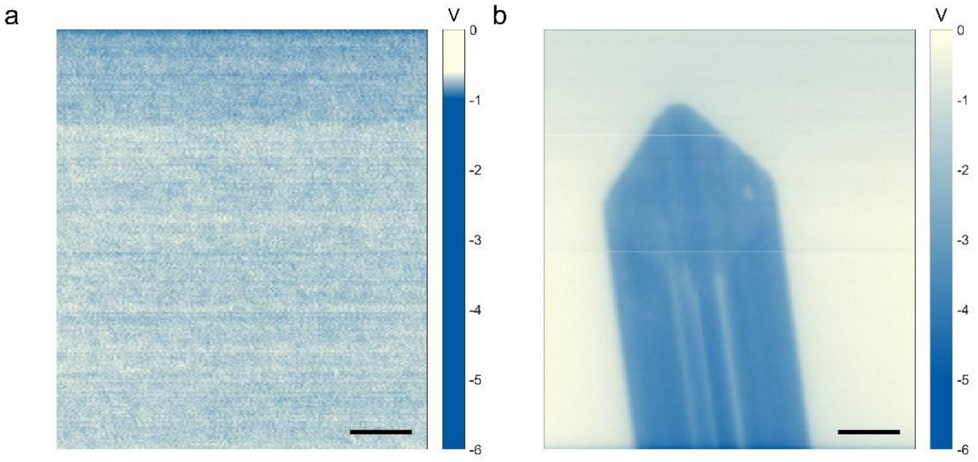

Figure S17. Surface potential of PTFE a) before and b) after tribocharging.

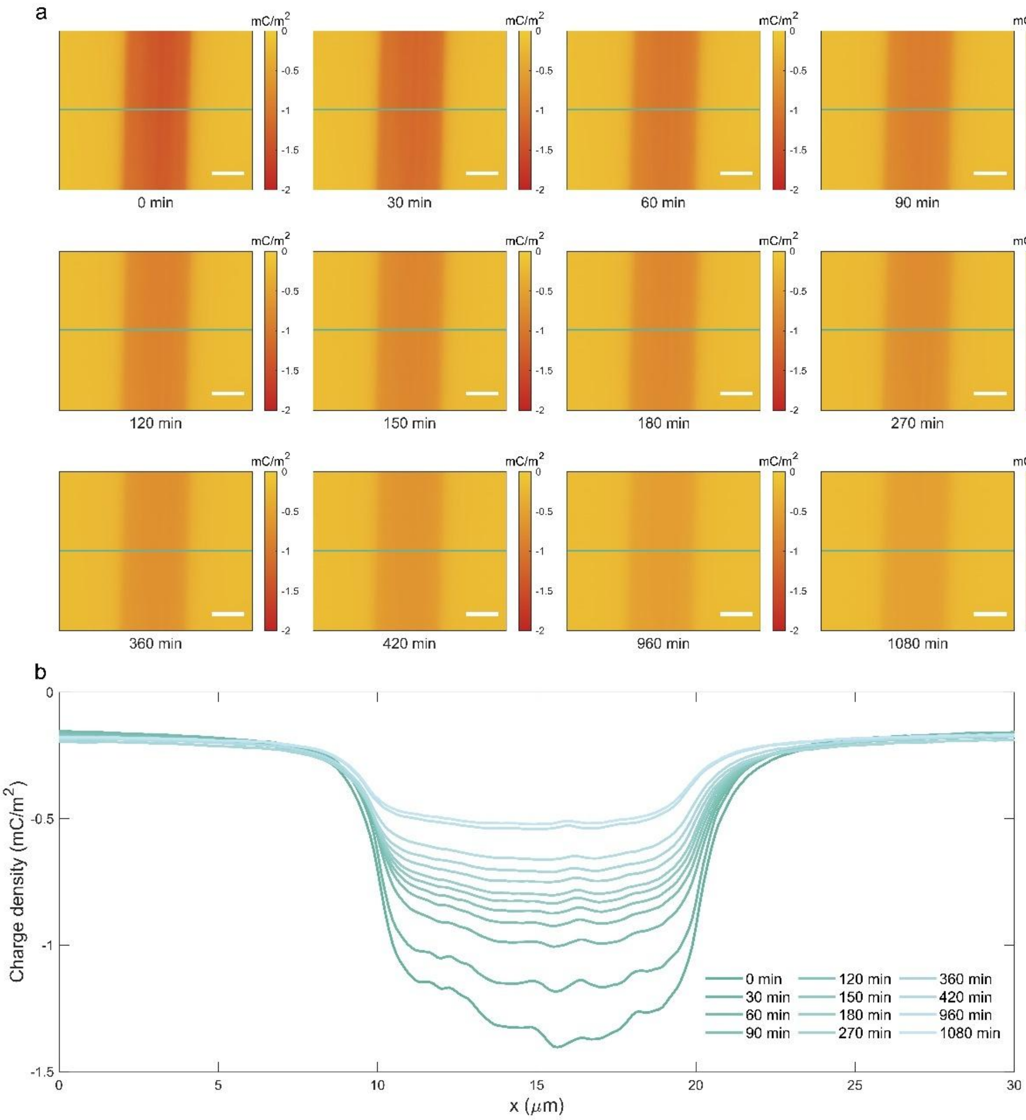

Figure S18. Charge decay of charged PTFE surface. a) Charge density distribution over time. Scale bar, 5 μm. b) Charge density decay curves.

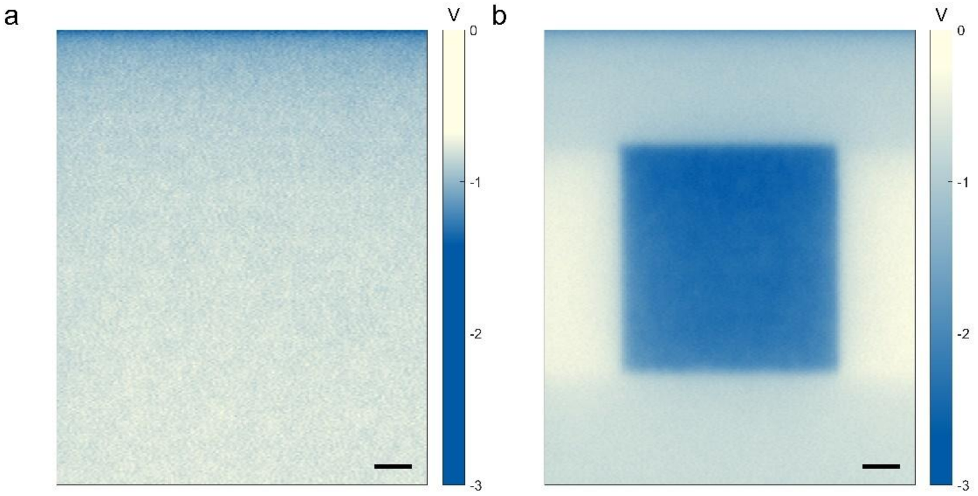

Figure S19. Surface potential of PTFE a) before and b) friction. Scale bar, 1 μm. AFM tip is used to conduct nanoscale contact electrification. The nanoscale contact electrification process is: first, using KPFM mode to scan the PTFE surface with a size of 10 × 10 μm^2; then, using AC mode to perform tribocharging in repulsive region (phase < 90°). The tribocharging area is 5 x 5 μm^2; Last, using KPFM mode to measure the surface potential with the scanning area of 10 × 10 μm^2. The middle area in (b) shows significant potential change compared with surrounding area.

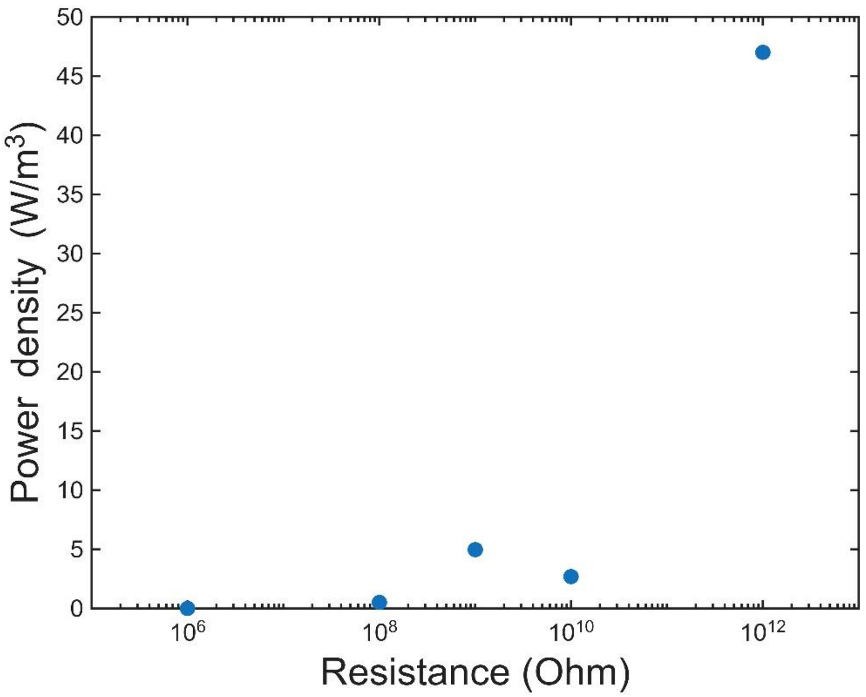

Figure S20. Power density of micro-TENG at different load resistances.

SUPPLEMENTAL NOTES

Note S1 Relative charge density extraction from KPFM

We use the typical capacitance model to describe the tribocharging behavior in PTFE, as described in Equation 1.

$$C_{Sample} = \frac{\varepsilon_0 \varepsilon_{Sample} A}{t_{Sample}} \tag{1}$$

C_{Sample} is the capacitance of dielectric sample, ε_0 is the vacuum dielectric constant, ε_{Sample} is the relative permittivity of sample, A is the contact area, t_{Sample} is the thickness of sample.

The capacitance of sample can also be expressed as:

$$C_{Sample} = \frac{Q}{V} \tag{2}$$

Combining Equation 1 and 2, we can get:

$$\sigma = \frac{Q}{A} = \frac{\varepsilon_0 \varepsilon_{Sample} V}{t_{Sample}} \tag{3}$$

The relative surface potential between charged area and uncharged area is used to represent the triboelectric charge transfer, as demonstrated in Figure S16. Therefore, the transferred charge density $\Delta\sigma$ can be calculated as:

$$\Delta\sigma = \frac{\varepsilon_0 \varepsilon_{Sample} \Delta V}{t_{Sample}} \tag{4}$$

Equation 4 calculates the relative charge density that reflects the charge transfer during CE. It cannot be used for absolute charge density calculation. The AFM data obtained from Cypher ES was processed in Matlab using the function '**IBWread**', which is included in '**Igor Pro file format (ibw) to matlab variable**' module. By converting AFM date file ('.ibw') to matrix, we can obtain the transferred charge density via matrix calculation.

Note S2 Van der Waals force simulation

In the microscale, the van der Waals (vdW) interaction cannot be ignored. To simulate the vdW force between the graphite microflake and PTFE surface, the Lennard-Jones (LJ) potential was used[1,2]. For qualitatively analyzing their interaction, we simulate the interaction between carbon atom and $(C_2F_4)_{10}$ molecule. So the LJ potential between them is given by:

$$V_{\mathrm{LJ}}(r) = 4\varepsilon_{12}\left[\left(\frac{\sigma_{12}}{r}\right)^{12} - \left(\frac{\sigma_{12}}{r}\right)^{6}\right] \quad (5)$$

where $\sigma_{12} = 3.629$ Å, and $\varepsilon_{12} = 0.205$ kcal/mol, r is the distance between two molecules. The interaction parameters σ_{12} and ε_{12} were determined by the combination rule:

$$\sigma_{12} = \frac{\sigma_1+\sigma_2}{2} \quad (6)$$

$$\varepsilon_{12} = \sqrt{\varepsilon_1 \times \varepsilon_2} \quad (7)$$

For carbon atom, $\sigma_1 = 3.55$ Å, $\varepsilon_1 = 0.07$ kcal/mol[3]; For $(C_2F_4)_{10}$, $\sigma_2 = 5$ Å, $\varepsilon_2 = 0.3$ kcal/mol[4]. The coordinate of carbon atom is (x_1, y_1, z_1) and that of $(C_2F_4)_{10}$ is (x_2, y_2, z_2), so the in-plane distance is:

$$r_{\mathrm{xy}} = \sqrt{(x_1 - x_2)^2 + (y_1 - y_2)^2} \quad (8)$$

And the distance r between them is:

$$r = \sqrt{r_{\mathrm{xy}}{}^2 + (z_1 - z_2)^2} \quad (9)$$

The total potential can be descried as:

$$V_{\mathrm{s}}(r) = \rho_1\rho_2 \int_{-\infty}^{0} \int_{r}^{\infty} \int_{0}^{\infty} V_{\mathrm{LJ}} 2\pi r_{\mathrm{xy}} dr_{\mathrm{xy}} dz_1 dz_2 = 4\varepsilon\rho_1\rho_2\pi\left(\frac{\sigma^{12}}{360r^8} - \frac{\sigma^6}{12r^2}\right) \quad (10)$$

where $\rho_1 = 1.14 \times 10^{29}\ \mathrm{m^{-3}}$ and $\rho_2 = 1.21 \times 10^{27}\ \mathrm{m^{-3}}$ are the atom number densities of carbon atom in graphite and $(C_2F_4)_{10}$ molecular in PTFE. The pressure of vdW interaction between graphite and silicon surfaces is:

$$P(r) = -\frac{dV_s(r)}{dr} = \varepsilon\rho_1\rho_2\pi\left(\frac{4\sigma^{12}}{45r^9} - \frac{2\sigma^6}{3r^3}\right) \tag{11}$$

The relationship between pressure P and distance r is plotted in Figure 3e.

Note S3 FEM simulation

The schematic diagram of the FEM simulation is shown in Figure 3d. The tungsten probe is simplified as a half sphere with a diameter of 4 μm. The size of W cap is 11.6 × 11.6 × 0.3 μm^3, and that of graphite microflake is 11 × 11 × 0.2 μm^3. The Young's module of W is 411 GPa; the Poisson ratio of W is 0.28. The graphite is orthotropic, and its elastic parameters are shown in Equation 12[5].

$$\begin{pmatrix} \sigma_x \\ \sigma_y \\ \sigma_z \\ \tau_{xy} \\ \tau_{yz} \\ \tau_{xz} \end{pmatrix} = \begin{bmatrix} 1060 & 180 & 15 & 0 & 0 & 0 \\ 180 & 1060 & 15 & 0 & 0 & 0 \\ 15 & 15 & 36.5 & 0 & 0 & 0 \\ 0 & 0 & 0 & 440 & 0 & 0 \\ 0 & 0 & 0 & 0 & 4.5 & 0 \\ 0 & 0 & 0 & 0 & 0 & 4.5 \end{bmatrix} \begin{pmatrix} \varepsilon_x \\ \varepsilon_y \\ \varepsilon_z \\ \gamma_{xy} \\ \gamma_{yz} \\ \gamma_{xz} \end{pmatrix} \quad (12)$$

where the unit is GPa. The size of the PTFE is 11.6 × 11.6 × 0.5 μm^3. To simulate the compression property of PTFE, a constitutive Equation 13 was used[6]:

$$\sigma = \sigma_Y\{1 - \exp\left[-(21.2 + 21.7\dot{\varepsilon}^{0.34})\varepsilon\right] + 4.04\varepsilon^{1.56}\} \quad (13)$$

where σ is the true stress, σ_Y is the yield stress, $\dot{\varepsilon}$ is the strain rate, ε is the true strain. For the values, σ_Y is set as 24.53 MPa and $\dot{\varepsilon}$ is 2000 s^{-1}. The true stress-true strain curves are shown in Figure S21. The simulation was performed on Abaqus 2024 with implicit solver. For investigating the size influence, the width of above parts was set in 5.8 μm, 7.1 μm, 9.5 μm, and 11.6 μm, and the loading force is 1000 μN. The loading force in Figure 3f and 3g is 1500 μN.

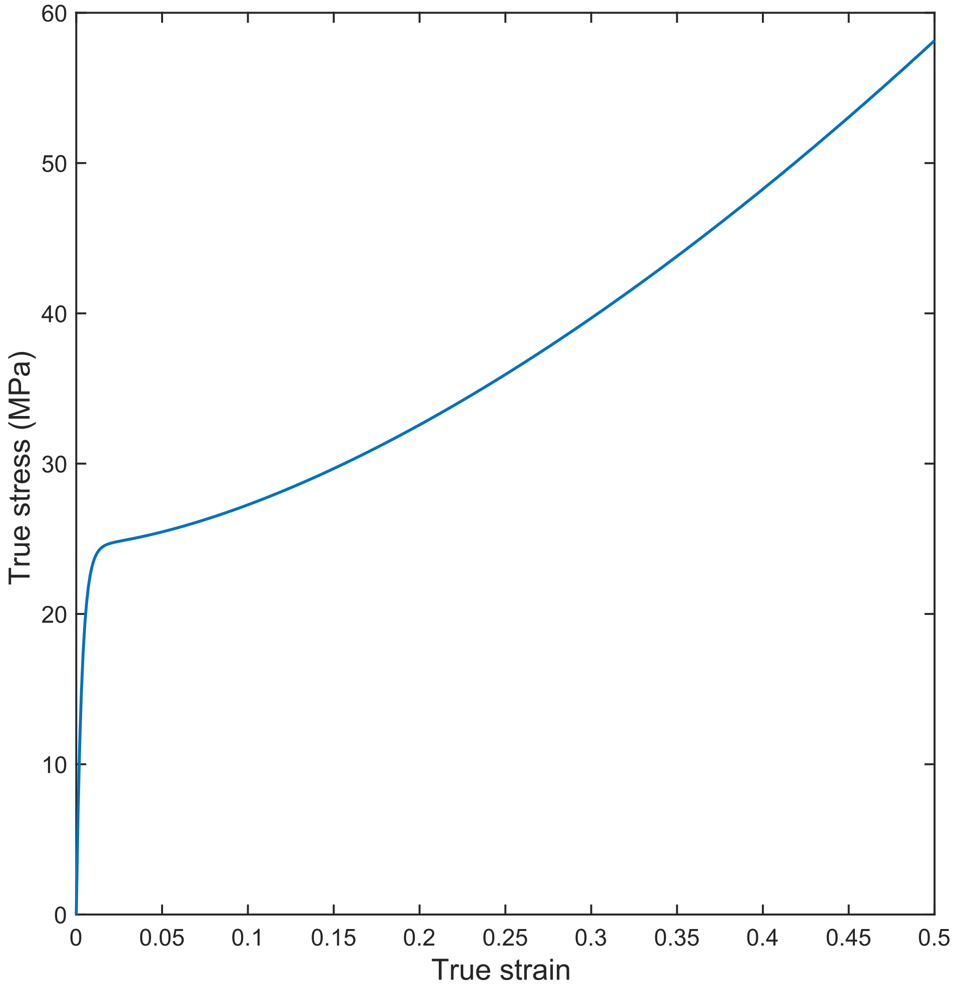

Figure S21. True stress-true strain curves of PTFE in compression condition. The blue line is obtained by constitutive equation.

Note S4 Estimating the capacitance of micro-TENG

The coplanar capacitor model was used to evaluate the capacitance of micro-TENG, as presented in Figure S22. The C_1 is induced by the fringing electric field, and the C_2 is induced by the lateral electric field. The C_1 can be expressed as[7,8]:

$$C_1 = \frac{2\varepsilon_r\varepsilon_0 l}{\pi}\ln\left[\left(1+\frac{w}{a}\right)+\sqrt{\left(1+\frac{w}{a}\right)^2-1}\right] \tag{14}$$

where ε_r is the relative dielectric constant, ε_0 is the vacuum permittivity, l is the length of electrode, w is the width of electrode, $2a$ is the gap distance between electrodes. The electric field has a penetration depth T[9],

$$T = a\sqrt{\left(1+\frac{w}{a}\right)^2-1} \tag{15}$$

In our model, $w = l = 10\,\mu m$, $a = 1.5\,\mu m$, ε_0 is 2.1 for PTFE. Substituting these parameters into Equation 15, the penetration depth T is 11.4 μm. So the thickness of PTFE is $t_1 = 90\,nm$ which is smaller than T. In this case, the capacitance is determined by the effective portion of electrodes, and the effective width w_{eff} is:

$$\frac{w_{\mathrm{eff}}}{a} = \sqrt{1+\left(\frac{t_1}{a}\right)^2}-1 \tag{16}$$

Finally, the C_1 can be calculated via w_{eff} and its value is 3.23×10^{-16} F.

The C_2 is induced by the lateral electric field coupling between two metal electrodes, which is:

$$C_2 = \frac{\varepsilon_r\varepsilon_0 l}{2a} \times t_1 \tag{17}$$

where t_1 is the thickness of the electrode. Substituting t_1 into Equation 17, the C_2 is equal to 5.58×10^{-18} F. Therefore, the capacitance of micro-TENG can be estimated as 3.28×10^{-16} F, which is much smaller than traditional macro-TENG (10^{-11} F level), making it challenging to measure the true voltage output of micro-TENG.

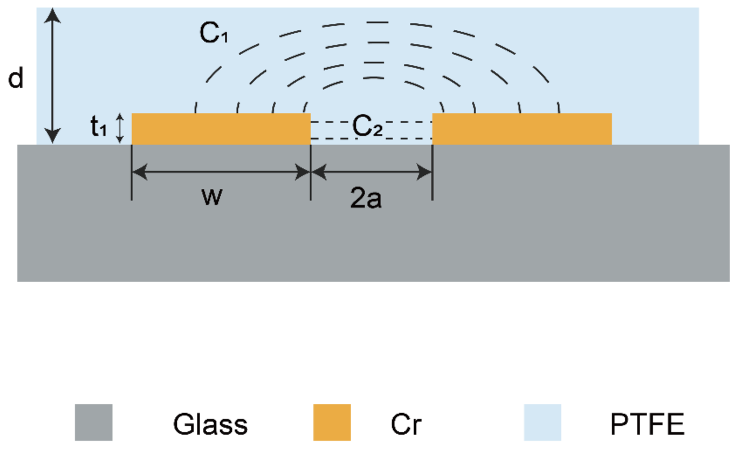

Figure S22. Capacitance model of micro-TENG.

Reference

1. Huang, X. *et al.* Robust microscale structural superlubricity between graphite and nanostructured surface. *Nat. Commun.* **14**, 2931 (2023).
2. Peng, D. *et al.* 100 km wear-free sliding achieved by microscale superlubric graphite/DLC heterojunctions under ambient conditions. *Natl. Sci. Rev.* **9**, nwab109 (2022).
3. Sinclair, R. C., Suter, J. L. & Coveney, P. V. Graphene–graphene interactions: friction, superlubricity, and exfoliation. *Adv. Mater.* **30**, 1705791 (2018).
4. Pan, D., Liu, C., Qi, X., Yang, Y. & Hao, X. A tribological application of the coarse-grained molecular dynamics simulation and its experimental verification. *Tribol. Int.* **133**, 32–39 (2019).
5. Wu, Z., Li, X., Peng, D. & Zheng, Q. Positive-negative tunable coefficients of friction in superlubric contacts. *Phys. Rev. Lett.* **132**, 156201 (2024).
6. Zhang, J., Ju, Y., Sun, C. & Wang, P. The research on compressive properties of polytetrafluoroethylene at high strain rate. *Def. Technol.* **9**, 181–185 (2013).
7. Martinez-Lopez, A. G., Guzmán-Caballero, D. E., Mejia, I. & Tinoco, J. C. Silicon based coplanar capacitive device for liquid sensor applications. *Sensors* **21**, 5958 (2021).
8. Dong, T. & Barbosa, C. Capacitance variation induced by microfluidic two-phase flow across insulated interdigital electrodes in lab-on-chip devices. *Sensors* **15**, 2694–2708 (2015).
9. Chen, J. Z., Darhuber, A. A., Troian, S. M. & Wagner, S. Capacitive sensing of droplets for microfluidic devices based on thermocapillary actuation. *Lab. Chip* **4**, 473 (2004).
